# Effects of Freeze-Dried Sake Lees and Rice Koji Extract on Osteoporosis in a Postmenopausal Mouse Model

**DOI:** 10.3390/nu17193077

**Published:** 2025-09-27

**Authors:** Jorge Sáez-Chandía, Stephanny Castillo-Quispe, Keiichiro Okamoto, Atsushi Kurahashi, Kazuya Kodaira, Kotaro Aihara, Kiyoko Suzuki-Barrera, Masaru Kaku, Yoshikazu Mikami, Miho Terunuma, Kensuke Yamamura, Takafumi Hayashi, Makio Saeki, Yoshito Kakihara

**Affiliations:** 1 Division of Oral and Maxillofacial Radiology, Faculty of Dentistry & Graduate School of Medical and Dental Sciences, Niigata University, Niigata 951-8514, Japan; jsaezch@gmail.com (J.S.-C.);; 2 Division of Dental Pharmacology, Faculty of Dentistry & Graduate School of Medical and Dental Sciences, Niigata University, Niigata 951-8514, Japan; stephanny.castillo@dent.niigata-u.ac.jp; 3 Faculty of Dentistry, Universidad de los Andes, Santiago 7580009, Chile; 4 Division of Oral Biochemistry, Faculty of Dentistry & Graduate School of Medical and Dental Sciences, Niigata University, Niigata 951-8514, Japan; 5 Division of Oral Physiology, Faculty of Dentistry & Graduate School of Medical and Dental Sciences, Niigata University, Niigata 951-8514, Japan; okamoto12@dent.niigata-u.ac.jp (K.O.);; 6 Sakeology Center, Niigata University, Niigata 950-2181, Japan; 7 Hakkaisan Brewery Co., Ltd., Minamiuonuma 949-7112, Japan; 8 Food Research Center, Niigata Agricultural Research Institute, Kamo 959-1381, Japan; 9 Division of Anatomy and Cell Biology of the Hard Tissue, Faculty of Dentistry & Graduate School of Medical and Dental Sciences, Niigata University, Niigata 951-8514, Japan; 10 Division of Bio-Prosthodontics, Faculty of Dentistry & Graduate School of Medical and Dental Sciences, Niigata University, Niigata 951-8514, Japan; 11 Division of Microscopic Anatomy, Graduate School of Medical and Dental Sciences, Niigata University, Niigata 951-8514, Japan

**Keywords:** sake lees, rice koji, osteoporosis, mouse model

## Abstract

**Background/Objectives:** With the aging of the population, the number of patients with osteoporosis is increasing worldwide. Osteoporosis results from an imbalance in bone remodeling by osteoblasts and osteoclasts. This study investigated the effects of sake lees and rice koji, traditional Japanese rice-fermented products, on bone metabolism. **Methods:** Both sake lees extract and rice koji extract increased alkaline phosphatase (ALP) activity, extracellular collagen accumulation, and mineralization of MC3T3-E1 cells. In addition, the intracellular protein levels of Hsp47 and Sec23IP, which are required for collagen maturation and secretion, respectively, were increased during the differentiation. On the other hand, both extracts significantly inhibited osteoclastic differentiation. Furthermore, the effects of freeze-dried sake lees or rice koji extract on osteoporotic bones were examined using twelve-week-old female C3H/HeJ ovariectomized (OVX) mice. **Results**: The groups of mice fed 20% or 40% freeze-dried sake lees showed significant suppression of the loss in bone volume fraction (BV/TV) and trabecular volume (Tb.V) compared with those fed a normal diet as well as the 40% freeze-dried sake lees-fed group reduced in the loss of trabecular thickness (Tb.Th). Similarly, the rice koji extract-treated mice showed significant inhibition of the loss in BV/TV, Tb.V, and even trabecular number (Tb.N.). Folic acid and S-adenosylmethionine (SAM), which have been reported to be present in sake lees, promoted extracellular collagen production by osteoblasts. **Conclusions**: In OVX mice, the intake of freeze-dried sake lees or rice koji extract was associated with the attenuation of trabecular bone loss, suggesting potential beneficial effects on bone metabolism.

## 1. Introduction

Bone is a highly dynamic mineralized connective tissue and is regulated by the action of osteoclasts, osteoblasts, osteocytes, and bone-lining cells [1]. Osteoclasts are responsible for bone resorption, whereas osteoblasts mediate new bone formation; the balanced activity of these cells is essential for maintaining skeletal strength, microarchitecture, and overall bone health [2]. However, an imbalance in the activity of these cells could adversely affect bone morphology, quality, and function, leading to bone metabolism diseases such as osteoporosis and osteopetrosis [3,4].

Osteoporosis is a systemic skeletal disease characterized by low bone mass and deterioration of the bone microarchitecture, leading to decreased mobility, chronic pain, and reduced quality of life, particularly in the elderly including postmenopausal women [5]. It is caused by an excessive increase in bone resorption [6]. Worldwide, osteoporosis is responsible for more than 8.9 million fractures annually, making this disease a serious public health challenge [7]. Its development can be caused not only by genetics, age, hormones, diseases, and certain medications, but also by lifestyle factors, with an unhealthy diet being a significant risk factor [8]. Therefore, a healthy and balanced diet is important for the proper growth and maintenance of bones, and a balanced intake of certain nutrients can be considered the first step in an effective preventive strategy for osteoporosis.

Fermented foods are increasingly recognized for their potential to support bone health, especially in vulnerable populations such as postmenopausal women. Yogurt consumption, for instance, has been positively associated with improved bone mineral density (BMD) in women, and in some cohort studies, with a reduced risk of hip fractures [9]. Also, fermented soy products such as natto are linked to lower bone loss at the hip and femoral neck and a reduced risk of osteoporosis in postmenopausal Japanese women [10,11].

The traditional Japanese diet is rich in rice, soybeans, and their various fermented products, such as sake (Japanese alcoholic beverage of fermented rice), miso (fermented soybean paste), shoyu (fermented soybean sauce), and mirin (sweet sake), which are fermented using koji mold (*Aspergillus oryzae*). Sake is made by fermenting rice with rice koji, which digests starch into sugars, which are then fermented by yeast (*Saccharomyces cerevisiae*) to produce ethanol. The solid material left after squeezing sake is called sake-kasu (sake lees), which contains not only carbohydrates, proteins, and fat, but also peptides, amino acids, vitamins, minerals, dietary fiber as well as microbial metabolic components such as folic acid and S-adenosylmethionine (SAM) [12,13]. As a by-product of sake production, sake lees represents an underutilized resource, and exploring their functional use can contribute to sustainable food utilization and valorization. Notably, folic acid and SAM are central components of one-carbon metabolism, which provides methyl groups for DNA and protein methylation in cells [14]. Impairment of one-carbon metabolism (e.g., folic acid deficiency leading to hyperhomocysteinemia) has been linked to osteoporosis and impaired bone formation [15,16].

So far, the health effects of sake lees include suppression of hypertension [17], allergic rhinitis [18], liver fat accumulation [19], psychophysical stress-induced hyperalgesia [20], and activation of odontoblastic cell differentiation [21]; the latter two were demonstrated in studies conducted by some of our coauthors. For rice koji, studies by the coauthors have reported improvements in defecation frequency [22], maintenance of skin moisture content [23], and suppression of anxiety- and pain-like responses under psychophysical stress conditions [24]. In addition, other studies have reported that rice koji lowers liver cholesterol [25]. The broad spectrum of these biological activities is likely attributable to the complex mixture of bioactive substances, including peptides, amino acids, polysaccharides, and microbial metabolites, present in the materials. Our research group, including coauthors of this manuscript, has conducted pioneering research on the biological effects of sake lees and rice koji. Given their potential to influence various physiological processes, we hypothesized that sake lees and rice koji may also exert beneficial effects on bone metabolism. To test this hypothesis, the objective of this study was to investigate the biological activity and mechanistic pathways of sake lees and rice koji on bone metabolism, utilizing both in vitro cellular experiments and in vivo validation with a postmenopausal osteoporosis mouse model.

## 2. Materials and Methods

### 2.1. Preparation of Sake Lees Extract and Rice Koji Extract

Sake lees was supplied by the sake brewery company (HAKKAISAN Brewery Co., Ltd., Niigata, Japan), which also financially supported this study. The funders had no role in the study design. First, sake lees was dissolved in a phosphate-buffered saline (PBS) buffer at a concentration of 0.5 g/mL, and then the solution was subjected to centrifugation for 30 min at 5000 rpm using a centrifuge (Kubota S500T, Kubota Corporation, Tokyo, Japan). The supernatant was obtained and filtered through a 0.22 µm pore size microfilter. This final product was used as the sake lees extract.

Dry rice koji was also supplied by the same company and ground using a coffee grinder (Varie Simple, Melitta Japan Co., Ltd., Tokyo, Japan), then mixed in PBS to a concentration of 0.5 g/mL. Subsequently, the solution was centrifuged for 30 min at a speed of 5000 rpm. The supernatant was filtered using a 0.22 µm pore size microfilter and used as the rice koji extract.

Basic compositional analysis was conducted for the prepared extracts. Protein content was determined by the combustion method, and the carbohydrate content was calculated by difference [100 − (moisture + protein + fat + ash)]. The 0.5 g/mL sake lees extract contained approximately 1 g protein and 7 g carbohydrate per 100 g, whereas the 0.5 g/mL rice koji extract contained approximately 0.4 g protein and 15.7 g carbohydrate per 100 g.

In this study, the sake lees extract was used only for in vitro experiments, while the rice koji extract was applied in both in vitro and in vivo experiments.

### 2.2. Cell Cultures

Murine pre-osteoblastic MC3T3-E1 cells and pre-osteoclastic RAW264.7 cells were maintained in a complete medium, composed of α-MEM supplemented with 10% fetal bovine serum (FBS), 100 units/mL penicillin, and 100 µg/mL streptomycin, at 37 °C under a humidified atmosphere of 5% CO_2_.

For the induction of osteoblastic differentiation, upon reaching 80–90% confluency, MC3T3-E1 cells were cultured in osteogenic differentiation medium, composed of the complete medium supplemented with 50 µg/mL ascorbic acid and 10 mM β-glycerol phosphate, and the cells were incubated at 37 °C under a humidified atmosphere of 5% CO_2_. The medium was replaced every 3 days. Sake lees extract or rice koji extract was added to the differentiation medium at a final concentration of 5 mg/mL. Similarly, folic acid (FUJIFILM Wako Pure Chemical Corporation, Osaka, Japan; purity ≥ 98%), S-adenosylmethionine (SAM) (Merck Millipore, Burlington, MA, USA; purity ≥ 80%), β-glucan (Merck Millipore, Burlington, MA, USA; purity ≥ 95%), chitin (Merck Millipore, Burlington, MA, USA; purity ≥ 75%), kojibiose (FUJIFILM Wako Pure Chemical Corporation, Osaka, Japan; purity ≥ 98%), mannan (Merck Millipore, Burlington, MA, USA; purity ≥ 98%), nigerose (Merck Millipore, Burlington, MA, USA; purity ≥ 98%), or α-ethylglucoside (α-EG) (FUJIFILM Wako Pure Chemical Corporation, Osaka, Japan; purity ≥ 98%) was added at a final concentration of 10 µg/mL.

For the induction of osteoclastic differentiation, RAW264.7 cells were cultured in the complete medium containing 100 ng/mL sRANKL (Oriental Yeast Co., Ltd., Tokyo, Japan) and incubated for 4 days at 37 °C under a humidified atmosphere of 5% CO_2_. Sake lees extract or rice koji extract was added to the differentiation medium at a final concentration of 5 mg/mL, 0.5 mg/mL, or 0.05 mg/mL.

### 2.3. Alkaline Phosphatase (ALP) Staining Assay

MC3T3-E1 cells were seeded in 24-well plates and cultured in osteogenic differentiation medium for 5 days. Then, the cells were fixed with 10% formalin solution for 10 min and incubated with SigmaFast^TM^ BCIP/NBT solution (Merck Millipore, Burlington, MA, USA) for 20 min at room temperature. After washing with distilled water, the wells were captured, and the stained areas were quantified using ImageJ software (version 1.54; National Institutes of Health, Bethesda, MD, USA).

### 2.4. Picro-Sirius Red Staining

Collagen matrix accumulation was assessed using MC3T3-E1 cells under the same culture conditions. After 5 days of osteogenic induction in 24-well plates, the cells were fixed with 10% formalin for 15 min and rinsed with PBS. The accumulated extracellular collagen was stained with the Picro-Sirius Red Stain Kit (ScyTek Laboratories, Inc., Logan, UT, USA) for 30 min at room temperature. After washing with distilled water, the stained dye was extracted by the extraction buffer (0.05 M NaOH and 50% methanol). The absorbance at 540 nm was quantified using a spectrophotometer (GloMax-Multi Detection System, Promega, Madison, WI, USA).

### 2.5. Alizarin Red S Staining

For mineralization assessment, MC3T3-E1 cells were maintained in osteogenic differentiation medium for 14 days in 24-well plates. The cultures were then fixed with 10% formalin solution for 10 min and washed with distilled water. Then, the deposited calcium was stained with 1% Alizarin Red S solution for 10 min at room temperature. After washing with distilled water, the wells were captured, and the stained areas were quantified using ImageJ software (National Institutes of Health, Bethesda, MD, USA).

### 2.6. Western Blot Analysis

The lysates were prepared with RIPA buffer (50 mM Tris HCl pH 8.0, 150 mM NaCl, 0.5% sodium deoxycholate, 0.1% sodium dodecyl sulfate, 1% Triton X-100) containing protease and phosphatase inhibitors (Thermo Fisher Scientific, Waltham, MA, USA) from cells collected in triplicate at 0, 3, 6, and 12 days after the induction of osteoblastic differentiation. The prepared samples were subjected to SDS-PAGE, and the separated proteins in the gel were transferred to a PVDF membrane (Pall Corporation, Port Washington, NY, USA), which was subsequently blocked with 5% *w*/*v* non-fat dry milk and incubated with primary antibodies at 2000-fold dilution, overnight at 4 °C. Anti-alkaline phosphatase tissue non-specific antibody (ab65834, Abcam, Cambridge, UK), anti-HSP47 antibody (GTX103011, GeneTex, Irvine, CA, USA), and anti-SEC23IP (p125) antibody (20892-1-AP, Proteintech, Rosemont, IL, USA) were used at 1:2000 dilution. Anti-β-actin antibody (010-27841, FUJIFILM Wako Pure Chemical Corporation, Osaka, Japan) was used at 1:10,000 dilution. Subsequently, the membranes were incubated with a secondary HRP-conjugated antibody, followed by a reaction with Western BLoT Hyper HRP Substrate (Takara Bio Inc., Shiga, Japan). The chemiluminescence image was obtained by ImageQuant LAS 4000 Mini (Cytiva, Tokyo, Japan).

### 2.7. Tartrate-Resistant Acid Phosphatase (TRAP) Staining and Cell Viability Assay for Osteoclastic Cells

Four days after the induction of osteoclastic differentiation in the presence of PBS, sake lees extract, or rice koji extract, RAW264.7 cells were fixed with 10% formalin at 37 °C for 15 min and subsequently incubated in TRAP buffer, composed of 0.1 M sodium acetate, 0.1 M acetic acid, 10 mg/mL Naphthol AS-MX phosphate, 0.1% Triton X-100, 0.3 M potassium tartrate, and 0.3 mg/mL Fast Red Violet LB Salt (Merck Millipore, Burlington, MA, USA) at 37 °C for 10 min. The TRAP-positive cells were identified and quantified using ImageJ software (National Institutes of Health, Bethesda, MD, USA).

The cell viability assay for RAW264.7 cells was performed using Cell Counting Kit-8 (Dojindo, Kumamoto, Japan). Cells were seeded in a 96-well plate at a density of 1 × 10^3^ cells/well and incubated in the complete medium for three days. In the presence of PBS, sake lees extract, or rice koji extract at 37 °C under a humidified atmosphere of 5% CO_2_, 10 μL of CCK-8 solution was added to the wells and incubated at 37 °C for two hours, and then absorbance at 450 nm was measured by a spectrophotometer (GloMax-Multi Detection System, Promega, Madison, WI, USA).

### 2.8. Animals and Experimental Design

All experiments and animal care were conducted in accordance with the ARRIVE guidelines [26] and followed the guidelines of the Japanese Animal Welfare and Handling Law (Law no. 105 of 1 October 1973). This study was conducted under the approval of the Intramural Animal Care and Veterinary Science Committee of Niigata University (Approval Number: SA00750, Approval Date: 17 August 2020; Approval Number: SA01276, Approval Date: 22 December 2022) following the established Guiding Principles for the Care and Use of Laboratory Animals (National Institutes of Health).

Twelve-week-old female C3H/HeJ mice were ovariectomized (OVX) or sham-operated at Japan SLC, Inc. (Shizuoka, Japan). Upon arrival, the mice were accommodated in transparent acrylic cages (30 × 20 × 15 cm) and provided with unrestricted access to a regular pelletized diet and water at 23 °C under a 12-h dark/light cycle. Mice were housed 3–4 per cage, and cage positions on each rack were rotated weekly to minimize the effect of light exposure. OVX mice were randomly assigned to each experimental group upon arrival to minimize potential baseline variability and were acclimatized for 4 days to allow them to adapt to the new environment. The total number of animals used in this study was 78. Animal use was minimized to ensure ethical and efficient experimental design.

Mice were either fed a diet containing freeze-dried sake lees in different proportions or orally administered rice koji extract. A positive control group received isoflavone. Effects were evaluated on the excised left distal femurs.

To test the effect of freeze-dried sake lees intake, all groups of mice were fed an AIN-93G based diet (Oriental Yeast Co., Ltd., Tokyo, Japan) [27]. Eight mice were obtained as sham-operated mice (sham, n = 8). The OVX mice were randomly divided into different groups: OVX mice fed a normal diet (control, n = 9), OVX mice fed 20% freeze-dried sake lees-containing diet (20% sake lees, n = 9), OVX mice fed 40% freeze-dried sake lees-containing diet (40% sake lees, n = 9), and OVX mice fed a 0.05% isoflavone containing diet (isoflavone, n = 9) as a positive control, and housed for 4 weeks (Table 1).

To examine the effect of rice koji extract intake, all groups of mice were fed the AIN-93G based diet [27]. Eight mice were obtained as sham-operated mice (sham, n = 8). The OVX mice were randomly divided into different groups: OVX mice orally applied water (control, n = 10), OVX mice orally applied 25 mg/mL rice koji extract (koji extract, n = 8), and OVX mice orally applied isoflavone containing water at a dose of 60 mg/kg daily (isoflavone, n = 8) as a positive control, and housed for 4 weeks (Table 2).

### 2.9. Micro-CT Analysis

The excised left distal femurs from mice were fixed with 4% formaldehyde and analyzed by micro-CT (CosmoScan FX; Rigaku Corporation, Tokyo, Japan) according to the manufacturer’s instructions, with an isotropic voxel size of 10 µm, voltage of 90 kV, and a current of 88 µA. A double filter consisting of 0.5 mm aluminum (Al) and 0.06 mm copper (Cu) was applied during scanning. One hundred and fifty cross-sectional images of the distal femoral metaphysis, starting at 0.2 mm from the end of the growth plate, were analyzed (corresponding to a total physical thickness of 3.0 mm; 150 slices × 0.02 mm per slice). The region of interest (ROI) was defined consistently in all samples to ensure comparable trabecular architecture measurements. Standard bone structure parameters including trabecular mineral density (Tb.BMD), trabecular volume (Tb.V), trabecular number (Tb.N), trabecular space (Tb.Sp), trabecular thickness (Tb.Th), bone volume fraction (BV/TV), bone volume (BV), tissue volume (TV), cortical mineral density (Ct.BMD), cortical volume (Ct.V), cortical thickness (Ct.Th), and total bone mineral density (total BMD) were analyzed using the Analyze 12.0 software (AnalyzeDirect, Inc., Overland Park, KS, USA). The micro-CT analysis was performed by an investigator blinded to group assignments.

### 2.10. Data Analysis

Statistical analyses were performed with EZR (Saitama Medical Center, Jichi Medical University, Saitama, Japan), which is a graphical user interface for R (The R Foundation for Statistical Computing, Vienna, Austria) [28]. All data were expressed as means ± standard deviation (S.D.). Normality of the data was assessed using the Shapiro–Wilk test, and homoscedasticity was evaluated by Levene’s test. For multiple comparisons, one-way analysis of variance (ANOVA) followed by Dunnett’s test was used to compare each group with the control group. Differences with *p* < 0.05 were considered significant. The examiners who conducted the statistical analysis were blinded to the respective treatment groups.

## 3. Results

### 3.1. Sake Lees Extract and Rice Koji Extract Promote Osteoblastic Differentiation of MC3T3-E1 Cells

**Figure 1:**
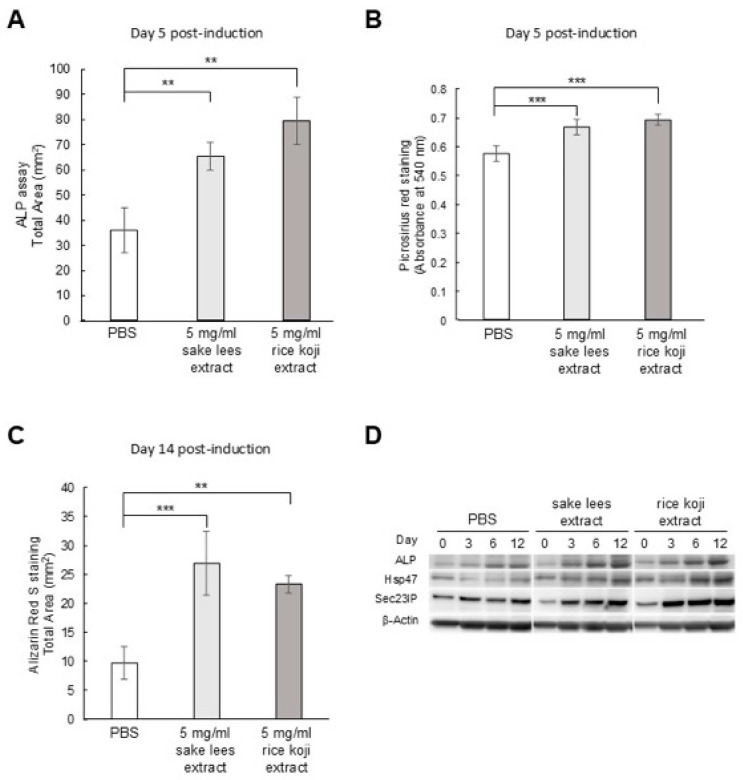
Effect of sake lees extract or rice koji extract on osteoblastic differentiation. MC3T3-E1 cells were cultured in osteogenic differentiation medium with PBS (control), 5 mg/mL sake lees extract, or 5 mg/mL rice koji extract, followed by the assays: (**A**) ALP staining, (**B**) Picro-Sirius red staining, and (**C**) Alizarin red S staining. Data were presented as mean values ± S.D. for ALP staining (n = 3), Picro-Sirius red staining (n = 4), and Alizarin red S staining (n = 4), and were analyzed using one-way ANOVA followed by Dunnett’s multiple comparison test. ** *p* < 0.01, and *** *p* < 0.001 *versus* PBS. (**D**) Western blot assay for ALP, Hsp47, and Sec23IP at days 0, 3, 6, and 12 during differentiation. β-Actin was used as an internal control.

To elucidate the biological activity of the sake lees extract and rice koji extract on osteoblastic differentiation, we investigated their effect on ALP activity, extracellular collagen accumulation, and mineralization using MC3T3-E1 cells. In the presence of 5 mg/mL sake lees extract or rice koji extract, the ALP levels were remarkably increased compared with the PBS control (Figure 1A and Figure S1A). Also, the accumulation of extracellular collagen, quantified by absorbance after Picro-Sirius red S staining, was increased with sake lees extract or rice koji extract treatment (Figure 1B). Similar results were observed in the Alizarin red S staining for mineralization (Figure 1C and Figure S1B). Furthermore, we examined the effect on the intracellular protein levels of ALP, Hsp47, and Sec23IP. Hsp47 is an essential molecular chaperone required for collagen maturation [29], and Sec23IP is involved in the collagen secretion pathway [30,31]. In the presence of sake lees extract or rice koji extract, the ALP, Hsp47, and Sec23IP levels were increased to higher degrees compared with the PBS control from 6 to 12 days after the induction of differentiation (Figure 1D). These results suggest that the sake lees extract and rice koji extract promote the osteoblastic differentiation of MC3T3-E1 cells and could promote the intracellular processes of collagen maturation and secretion.

### 3.2. Sake Lees Extract and Rice Koji Extract Inhibit Osteoclastic Differentiation of RAW264.7 Cells

**Figure 2:**
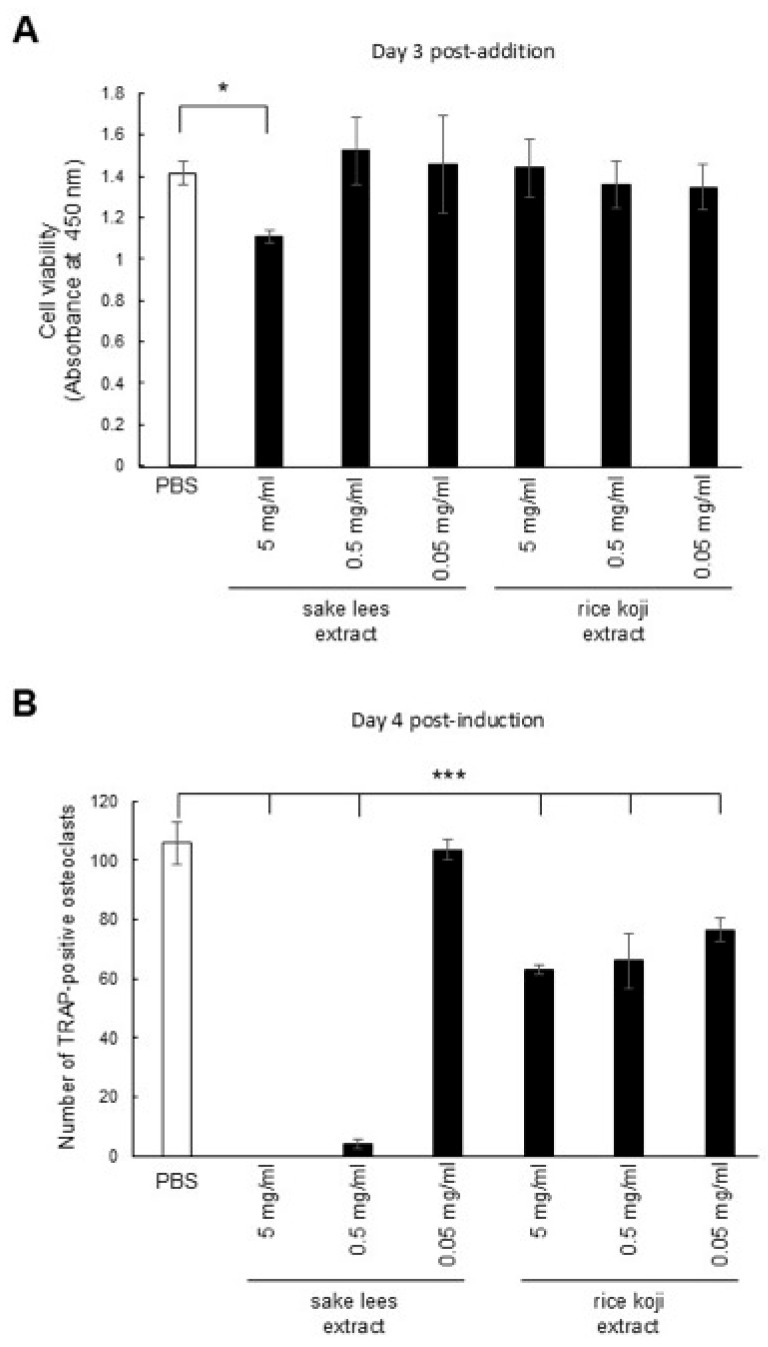
Effect of the sake lees extract or rice koji extract on osteoclastic cell viability and differentiation. (**A**) Cell viability assay for RAW264.7 cells treated with PBS (control), 5, 0.5, or 0.05 mg/mL sake lees extract, or 5, 0.5, or 0.05 mg/mL rice koji extract. Data are presented as mean values ± S.D. (n = 4) and were analyzed using one-way ANOVA followed by Dunnett’s multiple comparison test. * *p* < 0.05 *versus* PBS. (**B**) The number of TRAP-positive osteoclasts differentiated from RAW264.7 cells treated with PBS (control), 5, 0.5, or 0.05 mg/mL sake lees extract, or 5, 0.5, or 0.05 mg/mL rice koji extract. Data are presented as the mean values ± S.D. (n = 4) and were analyzed by one-way ANOVA followed by Dunnett’s multiple comparison test. * *p* < 0.05 and *** *p* < 0.001 *versus* PBS.

We examined the effect of the sake lees extract and rice koji extract on osteoclasts using pre-osteoclastic RAW264.7 cells. First, we evaluated the effects of the sake lees extract and rice koji extract on the cell viability at final concentrations of 5 mg/mL, 0.5 mg/mL, and 0.05 mg/mL. While the 5 mg/mL sake lees extract significantly decreased cell viability compared with PBS, the other concentrations of the sake lees extract as well as all concentrations of rice koji extract did not noticeably affect the cell viability (Figure 2A). Then, we examined their effects on osteoclastic differentiation. The number of mature TRAP-positive osteoclasts differentiated from RAW264.7 was significantly decreased by treatment with all samples, except for the 0.05 mg/mL sake lees extract, compared with PBS (Figure 2B). It should be noted that the decrease in TRAP-positive osteoclasts observed at the 5 mg/mL sake lees extract may be partially due to cytotoxic effects rather than solely to the inhibition of differentiation. These results show that both the sake lees extract and rice koji extract possess inhibitory activity against osteoclastic differentiation.

### 3.3. Intake of Freeze-Dried Sake Lees or Rice Koji Extract Maintains Bone Volume in OVX Mice

To investigate the effects of sake lees and rice koji on bone metabolism in vivo, we administered diets containing freeze-dried sake lees or rice koji extracts to OVX mice, a model of postmenopausal osteoporosis. It has been reported that C3H/HeJ mice exhibit human postmenopausal osteoporosis-like bone loss and cortical bone porosity after ovariectomy (OVX) in adults [32]. Therefore, C3H/HeJ mice were employed as a model of human postmenopausal osteoporosis in this study. To evaluate the efficacy of a dietary intervention with freeze-dried sake lees or rice koji extract initiated at the earliest possible stage following menopause, rather than after the progression of established osteoporosis, the experiment was designed to start the intake of samples immediately post-OVX surgery.

**Figure 3:**
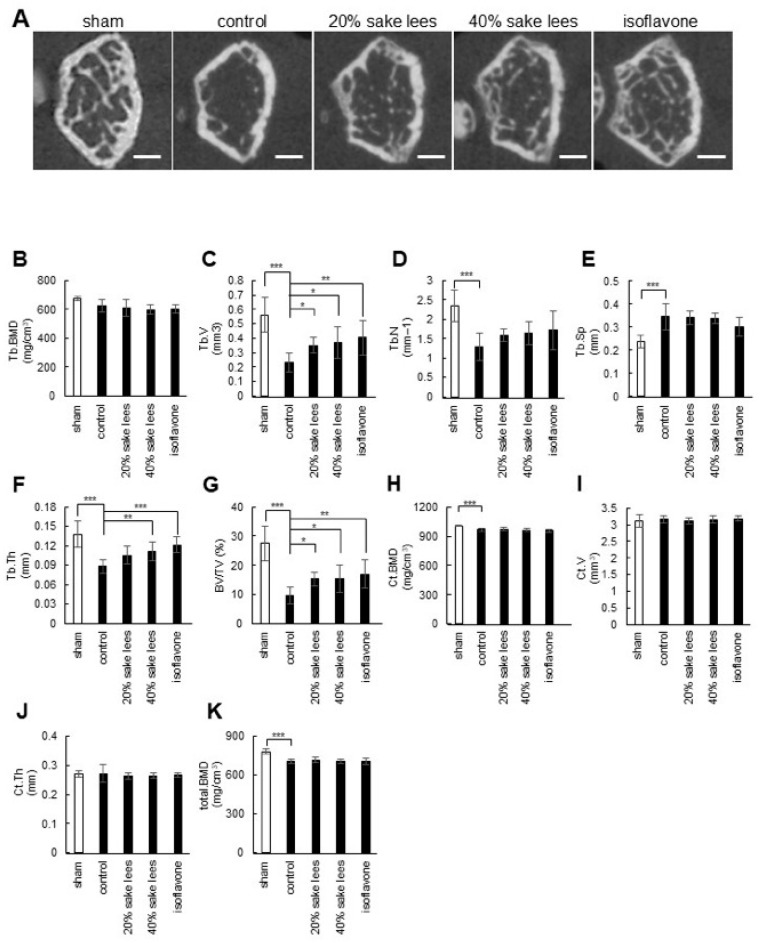
Effect of freeze-dried sake lees intake on the bone architecture of the distal femoral metaphysis in OVX mice. (**A**) Representative micro-CT images of each group: sham, control, 20% freeze-dried sake lees, 40% freeze-dried sake lees, and isoflavone. Scale bar: 0.5 mm. (**B**–**K**) Quantification of the bone microstructural parameters, Tb.BMD, Tb.V, Tb.N, Tb.Sp, Tb.Th, BV/TV, Ct.BMD, Ct.V, Ct.Th, and total BMD. Data are presented as the mean values ± S.D. (n = 8 for sham, n = 9 for control, n = 9 for 20% sake lees, n = 9 for 40% sake lees, and n = 9 for isoflavone) and were analyzed by one-way ANOVA followed by Dunnett’s multiple comparison test. * *p* < 0.05, ** *p* < 0.01, and *** *p* < 0.001 *versus* control.

First, to analyze the effect of freeze-dried sake lees, sham-operated mice were fed a normal diet, and OVX mice were fed a normal diet (control), a diet containing 20% freeze-dried sake lees or 40% freeze-dried sake lees, or a diet containing soy isoflavone as a positive control for 4 weeks. Then, the distal end of the left femur was captured using micro-CT (Figure 3A), and the bone microstructural parameters were analyzed (Figure 3B–K). The control OVX mice showed a decrease in Tb.V, Tb.N, Tb.Th, BV/TV, and total BMD, along with an increase in Tb.Sp compared with the sham-operated mice. Intake of 20% or 40% freeze-dried sake lees inhibited the decrease in Tb.V and BV/TV, and 40% freeze-dried sake lees inhibited the loss of Tb.Th compared with the control OVX mice (*p* < 0.05). Specifically, the decrease in Tb.V was inhibited by 52.2% (20% group) and 60.9% (40% group); the decrease in BV/TV was inhibited by 57.6% (20% group) and 58.7% (40% group); and the loss of Tb.Th was inhibited by 22.2% (40% group) compared with the control OVX mice.

**Figure 4:**
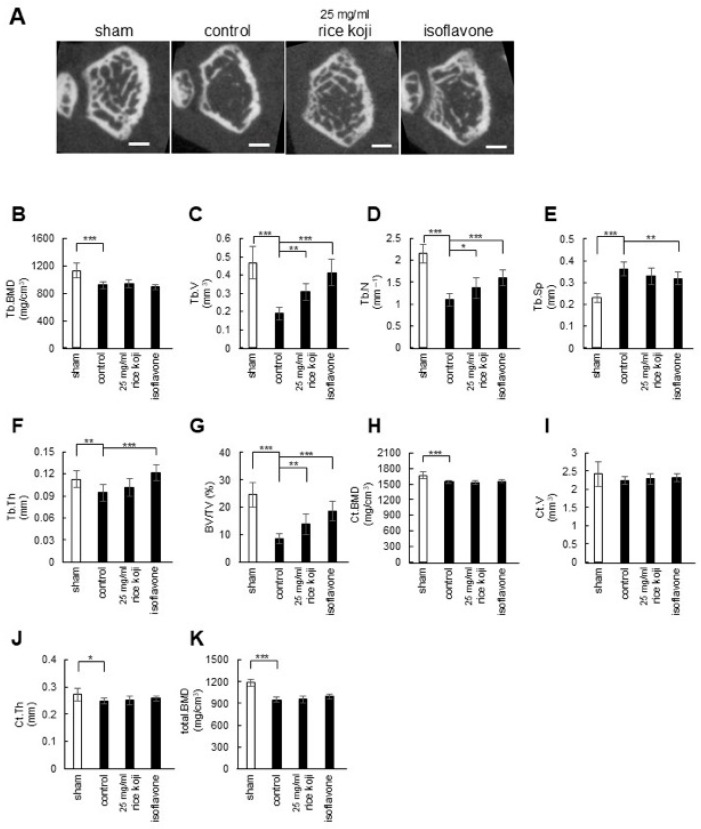
Effect of rice koji extract intake on the bone architecture of the distal femoral metaphysis in OVX mice. (**A**) Representative micro-CT images of each group: sham, control, rice koji extract, and isoflavone. Scale bar: 0.5 mm. (**B**–**K**) Quantification of the bone microstructural parameters, Tb.BMD, Tb.V, Tb.N, Tb.Sp, Tb.Th, BV/TV, Ct.BMD, Ct.V, Ct.Th, and total BMD. Data are presented as the mean values ± S.D. (n = 8 for sham, n = 10 for control, n = 8 for rice koji extract, and n = 8 for isoflavone), and were analyzed by one-way ANOVA followed by Dunnett’s multiple comparison test. * *p* < 0.05, ** *p* < 0.01, and *** *p* < 0.001 *versus* control.

Next, the effect of rice koji extract intake on osteoporotic bone in OVX mice was examined. All mice were fed a normal diet, with sham-operated mice receiving water, OVX mice receiving water (control), 25 mg/mL rice koji extract, or soy isoflavone-containing water for 4 weeks. Then, the distal end of the left femur was evaluated using micro-CT (Figure 4A), and the microstructural parameters were analyzed (Figure 4B–K). The administration of rice koji extract inhibited the decrease in Tb.V by 63.2%, Tb.N by 24.5%, and BV/TV by 60.5% compared with the control OVX mice. These results indicate that the intake of freeze-dried sake lees or rice koji extract did not markedly affect BMD in OVX mice, but effectively inhibited the loss of trabecular bone microstructure.

### 3.4. Investigation of Putative Active Substances in Sake Lees Extract and Rice Koji Extract on Osteoblastic Differentiation

**Figure 5:**
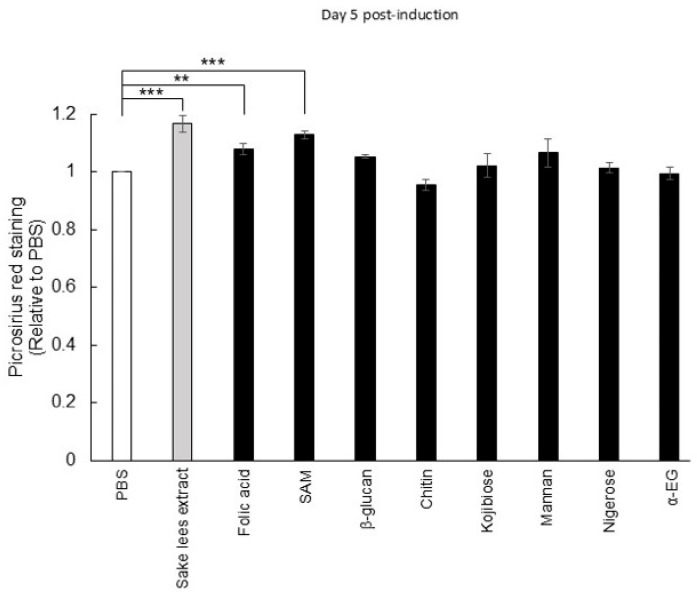
Effect of putative bioactive compounds in the sake lees extract or rice koji extract on osteoblastic differentiation. MC3T3-E1 cells were cultured in osteogenic differentiation medium with PBS, 5 mg/mL sake lees extract, 10 μg/mL folic acid, 10 μg/mL SAM, 10 μg/mL β-glucan, 10 μg/mL chitin, 10 μg/mL kojibiose, 10 μg/mL mannan, 10 μg/mL nigerose, or 10 μg/mL α-EG, followed by Picro-Sirius red staining. Data are presented as mean values ± S.D. (n = 4) and were analyzed by one-way ANOVA followed by Dunnett’s multiple comparison test. ** *p* < 0.01, and *** *p* < 0.001 *versus* PBS.

Based on the above results, it is suggested that sake lees extract and rice koji extract may contain potential bioactive compounds that stimulate bone formation. Therefore, we investigated the effects of compounds previously reported to be present in rice-fermented products, such as sake lees and rice koji, on the extracellular collagen accumulation of MC3T3-E1 cells. In this study, eight compounds were examined: folic acid, S-adenosylmethionine (SAM), β-glucan, chitin, mannan, 2-α-D-glucopyranosyl-D-glucose (kojibiose), 3-α-D-glucopyranosyl-D-glucose (nigerose), and ethyl α-D-glucoside (α-EG) [12,33,34,35,36,37]. Folic acid and SAM are produced by sake yeast during the sake brewing process [37]. β-Glucan, chitin, and mannan are derived from the cell walls of sake yeast and koji mold [12]. Kojibiose, nigerose, and ethyl α-D-glucoside (α-EG) are produced by the α-glucosidase of koji mold [33,34]. The results showed that 10 μg/mL folic acid or SAM significantly enhanced collagen accumulation (Figure 5). Although no significant effects were found, microbial cell wall components such as β-glucan and mannan also tended to promote the accumulation of collagen.

## 4. Discussion

In this study, we investigated the biological effect of sake lees extract, freeze-dried sake lees, and rice koji extract, derived from traditional Japanese rice-fermented products, on bone cells including osteoblasts and osteoclasts as well as in a mouse model of osteoporosis. In vitro, the sake lees extract and rice koji extract significantly promoted the ALP levels, collagen matrix accumulation, and mineralization in osteoblasts (Figure 1A–C). Additionally, increased levels of Hsp47 and Sec23IP were observed (Figure 1D). Hsp47 is an ER-resident molecular chaperone essential for the proper folding of triple-helical procollagen [38]. Sec23IP (or p125) is a Sec23p-interacting protein, a part of the COPII complex, facilitating transport from the ER to the Golgi [39]. It also helps form the tubular ER-Golgi intermediate compartment (tERGIC), a specialized carrier that efficiently transports large molecules, like the procollagen triple helix, from the ER to the Golgi [30]. Thus, these findings suggest that the sake lees extract and rice koji extract do not merely stimulate a general increase in extracellular collagen; rather, they appear to enhance the entire procollagen secretory pathway. The upregulation of Hsp47 expression likely improves the efficiency of procollagen folding and quality control within the ER. Concurrently, the observed increase in Sec23IP expression could be indicative of an augmented capacity for the vesicular transport of these bulky procollagen molecules from the ER to the Golgi via specialized tERGIC carriers. This coordinated activation of key components of the cellular protein synthesis and transport machinery may contribute to, or at least be associated with, the enhanced collagen matrix accumulation observed in osteoblasts following treatment with these extracts. We also examined the effects of the sake lees extract and rice koji extract on osteoclastic differentiation. Inhibition of differentiation was observed with the sake lees extract at 5 mg/mL and 0.5 mg/mL as well as with the rice koji extract at all concentrations (Figure 2B). For the sake lees extract at 5 mg/mL, the inhibition is likely due to the suppression of cell proliferation (Figure 2A). However, the sake lees extract at other concentrations and rice koji extract are suggested to have activity that inhibits osteoclastic differentiation.

Considering that an imbalance between osteoclastic bone resorption activity and osteoblastic bone formation activity leads to osteoporosis [40], our in vitro data are consistent with the hypothesis that sake lees extract or rice koji extract may have the potential to counteract osteoporosis. Therefore, we tested their inhibitory effects on osteoporosis progression using OVX model mice and found that freeze-dried sake lees intake inhibited the reduction in Tb.V, Tb.Th, and BV/TV compared with the control, with a greater effect observed at 40% freeze-dried sake lees than 20% freeze-dried sake lees (Figure 3). Similar effects were also detected with rice koji extract intake, showing significant inhibition of the decrease in Tb.V, Tb.N, and BV/TV (Figure 4). However, the magnitude of these effects was more modest compared with the intake of isoflavones, which are well-established phytoestrogens with bone-protective activity. One possible explanation for this modest effect is that freeze-dried sake lees and rice koji extracts represent complex mixtures, in which the concentrations of bioactive constituents may be relatively low. Furthermore, their activity may result from the combined actions of multiple compounds, not only folic acid and SAM, but also other unidentified bioactive molecules that may be present in the extracts. This interpretation is further supported by the results shown in Figure 5. Further studies will be necessary to clarify whether specific bioactive compounds within these extracts could contribute to stronger anti-osteoporotic effects.

Bone loss due to aging and/or menopause is known to initially manifest as the loss of trabecular bone, which includes trabecular thinning, disruption of the trabecular microstructure, and loss of trabecular elements. Subsequently, the cortical bone becomes more porous and reduced, and the intracortical surface increases [41,42]. In our in vivo experiments, after 4 weeks of OVX treatment, the control OVX group showed a significant reduction in trabecular bone mass, with minimal impact on cortical bone mass, compared with the sham group. This suggests that these mice could show early signs of osteoporosis. Since freeze-dried sake lees or rice koji extract intake primarily improved trabecular bone loss, they are likely to be effective in the early stages of osteoporosis (Figure 3 and Figure 4). Although the C3H/HeJ mouse strain has the advantages of earlier bone maturation, significantly higher bone density, and marked trabecular bone loss after ovariectomy compared with other strains (e.g., C57BL/6J), skeletal growth may still be underway in 12-week-old mice [43]. This may confound the assessment of bone loss due to menopause with the effects of ongoing bone formation. To eliminate potential bias due to ongoing skeletal growth, future studies should perform OVX in mice that have completed skeletal maturation and perform subsequent analyses. For example, performing surgery in skeletally mature animals (typically 16 weeks of age or older) and allowing for sufficient time (e.g., 4–8 weeks) after surgery to enable the trabecular bone loss to plateau would allow for the separation of estrogen deficiency-induced bone loss from normal growth and accumulation.

Our in vitro experiments showed a mineralization-promoting effect of the sake lees extract and rice koji extract; however, in vivo, this effect was not reflected in the recovery of bone mineral density. One possible explanation is that the experimental timeframe and dosing regimen may have been insufficient to elicit measurable changes in BMD. In addition, the mice were 12-week-old and still undergoing skeletal growth, which may have masked subtle effects of the samples on bone mass. Taken together, these factors could have contributed to the lack of detectable BMD improvements despite the in vitro mineralization-promoting activity of the extracts (Figure 2, Figure 3 and Figure 4). In addition, several limitations should be noted. First, the relatively small sample size may affect the generalizability of the findings. Second, the bioactive compounds in the sake lees and rice koji extracts were not fully standardized, which may affect the reproducibility and efficacy. Third, dynamic histomorphometry was not performed, limiting the ability to directly assess the bone formation and resorption rates. These constraints should be carefully considered when interpreting the in vivo results and planning future studies.

Sake lees and rice koji contain rice-derived proteins, carbohydrates, lipids, and dietary fibers as well as cellular components and metabolites derived from *koji* mold and *yeast* [12,33]. To identify the bioactive compounds for osteoblastic differentiation, we examined eight of the known components contained in rice-fermented foods such as folic acid, SAM, β-glucan, chitin, kojibiose, mannan, nigerose, and α-EG. A significant increase in collagen accumulation was observed with folic acid and SAM (Figure 5). It should be noted that we did not directly measure the presence or concentration of these compounds in the extracts used in this study due to limitations in our analytical facilities. Instead, their effects were examined using commercially obtained standards. Based on reports in the literature, sake lees contain approximately 3 mg/g of SAM and 2 µg/g of folic acid [13,36]. However, because we used extracts rather than whole sake lees, the actual concentrations of these compounds in the extracts are unknown. Nevertheless, the results from our cell culture experiments suggest that these bioactive compounds may be present at levels sufficient to elicit biological effects. Folic acid and SAM are involved in a process called one-carbon metabolism, interconnected biochemical pathways that generate methyl groups [44]. In this context, folic acid is crucial for the synthesis of purines and thymidines, necessary for DNA replication and cell division. It is also involved in regulating the homeostasis of specific amino acids including glycine, serine, and methionine [45]. Note that studies on humans have shown a reduced risk of osteoporosis with folic acid intake [16,46]. Given that sake lees contain approximately 200 µg of folic acid per 100 g [13], the intake levels employed in these human studies would correspond to consuming roughly 200 g of sake lees per day. While this amount may be achievable, it could be impractical as a daily dietary intake, and thus further studies are warranted to evaluate feasible consumption levels for bone health benefits. SAM is synthesized from methionine by methionine adenosyltransferase in one-carbon metabolism and functions as a universal methyl group donor. It provides methyl groups to various biomolecules such as DNA, RNA, and proteins. Therefore, SAM is involved in epigenetic regulation, contributing to DNA methylation and histone modifications, influencing gene expression and cellular differentiation [47]. Intriguingly, SAM has been shown to promote osteoblastic differentiation by activating SAM-dependent methyltransferases and enhancing the transcriptional activity of Runx2, a key regulatory transcription factor for differentiation [48]. Furthermore, a recent study suggests that one-carbon metabolism is activated during osteogenic differentiation, and conversely, its suppression could lead to impaired bone formation [49]. Therefore, it is suggested that folic acid and SAM present in sake lees may enhance one-carbon metabolism involved in osteoblast differentiation and thereby suppress the progression of osteoporosis. In contrast, there have been no reports indicating the presence of these compounds in rice koji, implying the involvement of other bioactive substances. Moreover, these rice-derived fermented foods likely contain additional bioactive components that were not examined in the present study such as phenolic acids, peptides, and organic acids [12,13,50]. Thus, further investigations are warranted to identify and characterize these active ingredients.

It should be noted that in this study, we primarily tested extracts or freeze-dried preparations rather than whole foods, so the effects observed may differ from consuming sake lees or rice koji directly. Nevertheless, these findings highlight the potential benefits of sake lees for bone health and underscore their value as a sustainable resource. By utilizing sake lees, a by-product of sake production, this study demonstrates a practical approach for the valorization of traditional fermented foods, contributing to sustainable food systems. Research on the health benefits of traditional Japanese fermented rice products is still in its early stages, and future studies may reveal their connection to the health of the Japanese population.

## 5. Conclusions

This study investigated the effects of sake lees extract, freeze-dried sake lees, and rice koji extract, derived from traditional Japanese rice-fermented foods, on osteoblasts, osteoclasts, and a mouse model of osteoporosis. The results demonstrated that both the sake lees extract and rice koji extract stimulated osteoblastic activity while suppressing osteoclastic function in vitro. In addition, freeze-dried sake lees or rice koji extract attenuated the loss of trabecular bone volume in the femurs of OVX mice over four weeks. Furthermore, folic acid and S-adenosylmethionine (SAM), which have been reported to be present in sake lees, were found to promote collagen production in osteoblasts in our cell culture experiments, suggesting a potential mechanism underlying their bone-preserving effects.

## Figures and Tables

**Table 1:** Nutrient composition of AIN-93G and freeze-dried sake lees diets (per 100 g).

Component	AIN-93G	20% Freeze-Dried Sake Lees	40% Freeze-Dried Sake Lees
Protein (g)	21.5	18.9	16.3
Lipid (g)	6.1	5.4	4.7
Carbohydrate (g)	54.6	51.6	48.6
Energy (kcal)	359.8	340.8	321.9

**Table 2:** Nutrient composition of rice koji extract (per 100 g).

Component	25 mg/mL Rice Koji Extract
Protein (mg)	20
Lipid (mg)	5
Carbohydrate (mg)	785
Energy (kcal)	3.2

## Data Availability

The original contributions presented in this study are included in the article/Supplementary Materials. Further inquiries can be directed to the corresponding author.

## Supplementary Materials

The following supporting information can be downloaded at: https://www.mdpi.com/article/10.3390/nu17193077/s1, Figure S1. Effect of sake lees extract or rice koji extract on osteoblastic differentiation: ALP activity and mineralization.
