# Supplementary figures and images for: Effects of Freeze-Dried Sake Lees and Rice Koji Extract on Osteoporosis in a Postmenopausal Mouse Model
Source: Nutrients. 2025 Sep 27;17(19):3077. doi: 10.3390/nu17193077 (PMC12525797; doi:10.3390/nu17193077)

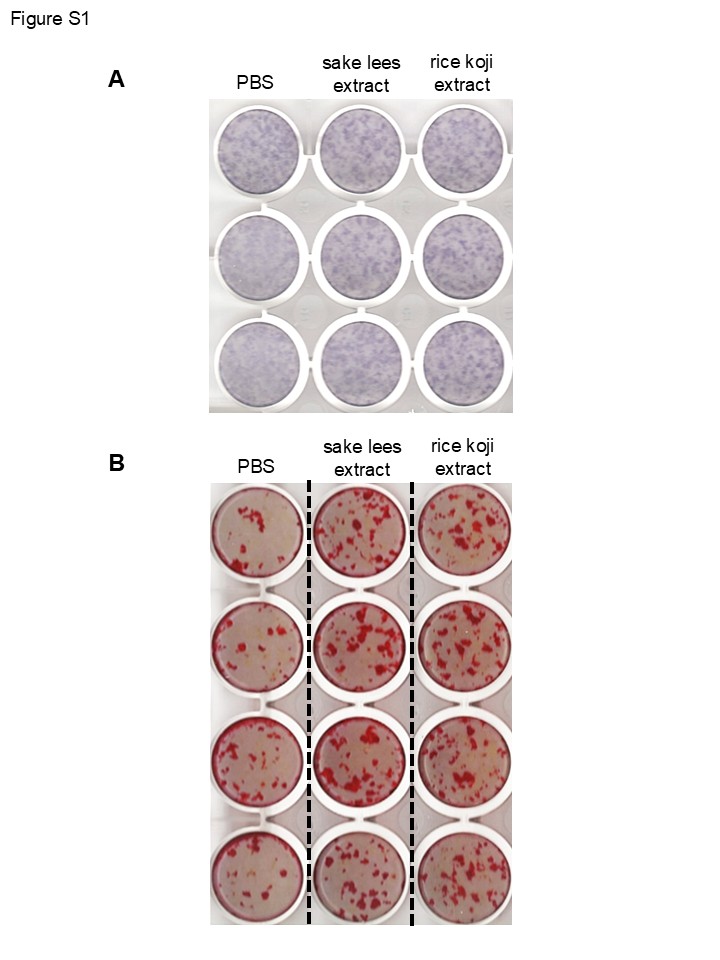

Supplement: Supplementary file 1 [file nutrients-17-03077-s001.zip › nutrients-3805050-supplementary.jpg]
